# Disruption of the structural maintenance of chromosomes 5/6 complex enables tumor mutagenesis

**DOI:** 10.1093/narcan/zcag012

**Published:** 2026-05-21

**Authors:** Thi Tran, Jiayi Fan, Xiaolan Zhao, Abby M Green

**Affiliations:** Department of Pediatrics, Washington University School of Medicine, St. Louis, MO 63110, United States; Center for Genome Integrity, Siteman Cancer Center, Washington University School of Medicine, St. Louis, MO 63110, United States; Department of Molecular Biology, Memorial Sloan Kettering Cancer Center, NY, NY 10065, United States; Department of Molecular Biology, Memorial Sloan Kettering Cancer Center, NY, NY 10065, United States; Department of Pediatrics, Washington University School of Medicine, St. Louis, MO 63110, United States; Center for Genome Integrity, Siteman Cancer Center, Washington University School of Medicine, St. Louis, MO 63110, United States

## Abstract

The structural maintenance of chromosomes (SMC) 5/6 complex is conserved and essential for mammalian development. SMC5/6 germline variants in cells, model organisms, and patients results in genome instability. However, the consequences of somatic SMC5/6 dysfunction in cancer are unknown. We report a pan-cancer analysis of SMC5/6 variants across three databases in which thousands of tumors across all tissue types harbored copy number alteration (CNA) and/or small variants in SMC5/6 genes. We found that deleterious variants—those predicted to cause protein disruption, but not CNAs, in SMC5/6 genes were associated with elevated tumor mutational burden (TMB). Mutagenesis in tumors with deleterious SMC5/6 variants was largely due to polymerase epsilon dysfunction and mismatch repair deficiency. Patients with SMC5/6 gene variants in tumors exhibited improved survival, in part due to a superior response to immunotherapy. Our findings demonstrate that SMC5/6 complex disruption in cancer predicts elevated TMB and susceptibility to immunotherapy, indicating the potential to use SMC5/6 gene status for prognostic implications and tailored therapeutic approaches.

## Graphical Abstract

**Graphical Abstract:**
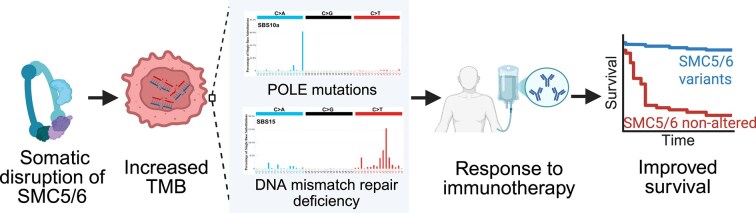

## Introduction

The three eukaryotic structural maintenance of chromosomes (SMC) complexes, condensin, cohesin, and SMC5/6, are highly conserved and play important roles in genome integrity [1]. The complexes are structurally similar, comprised of dimers of two distinct SMC proteins that can form elongated rings or filamentous structures, as well as accessory proteins [1]. SMC5/6 is distinct in that it contains protein-modifying subunits. Two of its non-SMC element (NSMCE) accessory subunits include the SUMO ligase (E3), NSMCE2, and a putative ubiquitin ligase, NSMCE1 [2, 3]. While cohesin and condensin are well known to organize chromatin by generating DNA loops [4, 5] (reviewed in [4] and [5]), the function of SMC5/6 appears to be more diverse and remains enigmatic [4, 6]. The human SMC5/6 complex is comprised of six interacting proteins (SMC5, SMC6, NSMCE1–4) as well as the SLF1 and SLF2 subunits. Human SMC5/6 is best defined for its role during interphase to aid in recombinational DNA repair, replication completion, and coping with replication stress [7–12]. These functions are also assigned to the yeast Smc5/6, which can interact with double-stranded and single-stranded DNA gaps and generate DNA loops *in vitro* [13–23].

Recently, germline SMC5/6 gene alterations have been reported in several patients with severe developmental diseases. Four studies over the last decade have detailed patients with biallelic mutations in SMC5 [24, 25], NSMCE2 [26], NSMCE3 [27], and the chaperone SLF2 [24]. All patients had microcephaly and developmental defects with some manifesting additional phenotypes including severe lung disease, dwarfism, insulin resistance, and bone marrow failure. While some variability exists among the syndromes reported, the patients consistently displayed DNA damage and genome instability as evidenced by abnormal chromosomes, micronuclei, and nuclear bridges [24–27]. In comparison with these established effects of germline SMC5/6 dysfunction, the effect of SMC5/6 dysfunction in cancer is still an open question.

In a pan-cancer study, SMC5 and SMC6 were found to be among the top 50 most frequently mutated DNA damage genes [28]. Despite this frequency, a recent evaluation of SMC5/6 gene alterations in breast cancer is the only published study linking somatically-acquired abnormalities of the complex to tumor biology [29]. In this study of breast cancer data collected from cBioPortal, alteration of copy number or DNA sequence in any SMC5/6 gene was associated with increased aneuploidy score and poor overall survival [29]. With this link as a foundation, we sought to define the effect of somatic SMC5/6 dysfunction across human cancers.

We undertook an *in silico* study to define the frequency and impact of SMC5/6 disruption in human cancer. We examined three large datasets of annotated cancer genomes, namely The Cancer Genome Atlas (TCGA), Pan-Cancer Analysis of Whole Genomes (PCAWG), and Colorectal Cancer Whole Genome Sequencing (Hartwig Medical Foundation). We found that a large fraction of tumors had alterations in SMC5/6 complex genes, either through copy number changes or small sequence variants (base substitutions, insertions/deletions). We found that small variants (SmVs) were broadly distributed across SMC5/6 complex genes rather than at recurrent “hotspot” sites. This nonspecific variant pattern is consistent with a pattern we identified in other cancer-related complexes. SmVs, in comparison to copy number alteration (CNA), were associated with increased tumor mutational burden (TMB) and mutagenesis caused by polymerase epsilon (POLE) defects and mismatch repair deficiency (MMRd). Importantly, we found SmV in SMC5/6 genes are associated with improved responses to immunotherapy. Together our data demonstrate the impact of deleterious variants of SMC5/6 on cancer genomes, a strong association between SMC5/6 variants and POLE/MMRd-mediated mutagenesis, and implications for use of SMC5/6 gene status as a biomarker for response to immune checkpoint blockade.

## Materials and methods

### SMC5/6 gene alteration identification and cohort analysis

Our study includes 9614 samples from TCGA, 2 778 samples from PCAWG, and 724 samples from Hartwig Medical Foundation. Only primary samples from the TCGA and PCAWG projects were included in this study. Data obtained from the Hartwig Medical Foundation consisted of metastatic colorectal cancer genomes [30]. Samples missing data from somatic variant calling, CNAs, or survival information were eliminated from cohort analysis. The final dataset contained 8809 TCGA samples, 1396 PCAWG samples, and 545 Hartwig samples. Hartwig variant calling files were processed with VEP (release 112.0) and vcf2maf (https://github.com/mskcc/vcf2maf) to predict mutation consequences. SmVs with either HIGH VEP impact [31] or SIFT score <0.05^32^ were predicted to be deleterious. Tumors were selected for exonic SmVs in at least one of the SMC5/6 genes (SMC5, SMC6, NSMCE1, NSMCE2, NSMCE3, NSMCE4A, NSMCE4B, SLF1, and SLF2), and classified as deleterious, synonymous, or NS/ND. The sorting order for these cohorts were deleterious, NS/ND, and synonymous to account for samples with multiple types of SMC5/6 SmVs. Tumors were also selected for having abnormal copy number (≠ 2) of at least one of the SMC5/6 gene segments. For both TCGA and PCAWG data, low copy number variants (low CNA) cohort consisted of tumors with copy number <2, while high CNA cohort consisted of those with copy number >2. With Hartwig data, low CNA group contained samples with maxCopyNumber ≤1.5, and high CNA group were made up of tumors with minCopyNumber ≥2.5. Samples with both low and high CNA of SMC5/6 genes were eliminated from later analysis. Samples with both SMC5/6 SmVs and abnormal copy number of SMC5/6 genes segments were included in both SmV and CNA groups. Samples with neither of these genetic alterations in any of the SMC5/6 genes were assigned to the nonaltered group. As there were not many nonaltered samples identified in the Hartwig dataset, abnormal CNA and nonaltered cohorts were grouped together in later analysis (labelled as “SmV absent”).

### Co-occurrence of SMC5/6 alterations, SmV type and frequency, and variant allele frequency

Tumors with multiple SMC5/6 alterations were identified. The sorting order (deleterious, NS/ND, and synonymous) was removed in this analysis to best reflect the number of tumors that carry each type of SMC5/6 SmV. Co-ocurrence of alterations (CNA and/or SmV) across SMC5/6 genes was depicted using package UpSetR (version 1.4.0) in R. Mutation frequency was calculated using the equation below:


\begin{eqnarray*}
{\mathrm{ Mutation}}\, {\mathrm{ frequency}} = \frac{{ {\mathrm{ Number}} \ \mathrm{ of} \ {\mathrm{ SmVs}}}} {{ {\mathrm{ Gene}} {\mathrm{ size}}}}
\end{eqnarray*}

where number of SmVs was the total number of mutation events of a SMC5/6 gene identified in TCGA. Variant allele frequency was calculated as


\begin{eqnarray*}
\mathrm{ VAF} = \frac{{{{n}_{{\mathrm{ alternate}}}}}}{{{{n}_{{\mathrm{ alternate}}}} + {{n}_{{\mathrm{ reference}}}}}}
\end{eqnarray*}

where ${{n}_{{\mathrm{ alternate}}}}$ was the number of reads with the altered base, and ${{n}_{{\mathrm{ reference}}}}$was the number of reads with the reference base at the locus.

### SMC5/6 SmV mapping and yeast homology assessment

All deleterious SMC5/6 variants identified in TCGA were mapped by protein location and resulting changes in amino acids sequence. The frequency of variant occurrence in TCGA was then counted and visualized with a lollipop chart generated using ggplot2 R package (version 4.0.0). Cancer-associated SMC5/6 SmV were aligned with each SMC5/6 subunit annotated with its known conserved structural and functional domains. Deleterious truncating mutations and single amino acid substitutions were considered for homology analysis. Mutations located in unstructured regions were not analyzed due to insufficient model reliability. AlphaFold was used to predict models of the human SMC5/6 arm and EID3 helix–turn–helix (HTH) domain and its interface with part of the SMC6 arm.

#### Sequence alignment

Sequences were obtained from UniProt [33], and multiple sequence alignment was performed using Clustal Omega [34] with default parameters. Alignment results are visualized using the ESPript3 website online tool [35]. Predicted pathogenicity scores were obtained from AlphaMissense Website [36] (https://alphamissense.hegelab.org).

#### Protein structure predictions and analyses

Structure predictions were performed using AlphaFold3 [37], pLDDT (0–100), pTM (0–1), and ipTM (0–1) are presented in the figure legend. All models were visualized in UCSF ChimeraX [38] and the Rotamer tools were used to obtain the model with mutation. Analyses of surface and atom interactions were performed using default setting in ChimeraX.

### TMB, aneuploidy, and mutational signature analysis

#### TMB and aneuploidy score analysis

TMB (total number of mutations per Mb) was calculated as the number of all SmV in a tumor, divided by either 30 (for TCGA exomes) or 2 800 (for PCAWG and Hartwig genomes). Silent and intronic SmVs were included in this calculation. Pairwise comparisons of TMB between cohorts were conducted and visualized using R package ggpubr (version 0.6.0). The relationship between number of SMC5/6 SmVs in each tumor and corresponding TMB was visualized as a linear regression model and evaluated with Pearson correlation. To analyze the distribution of SMC5/6 SmVs with different predicted outcomes across the TMB spectrum, sorting bias (deleterious > NS/ND > synonymous) was removed. The distribution was evaluated with Chi-square test. TCGA aneuploidy scores and PCAWG ploidy scores were readily available in cBioPortal (see the ‘Data availability’ section).

#### Single-base-substitution signatures analysis

Trinucleotide context of each tumor was generated by counting the number of single-base substitution (SBS) events (C > T, C > A, C > G, T > C, T > A, T > G), along with the 5′ and 3′ adjacent base. The mutational contexts of different samples were then added together to form a single trinucleotide context matrix that represented the study cohorts to which they belonged. These matrices were refitted to COSMIC Single-base-substitution signatures (SBSsig; version 3.2) using R package MutationalPatterns (version 3.14.0). The result was a combination of established COSMIC SBSsig that best reconstructed the mutational matrix of each cohort.

#### Analysis of tumors with co-mutated SMC5/6, POLE, and MMR genes

TCGA exomes were selected for nonsynonymous SmV in at least one of the POLE (POLE, POLE2, POLE3, POLE4) or mismatch repair (MMR; MLH1, MLH2, MLH3, MSH2, MSH6, PMS1, MS2) genes. Tumors were further categorized by nonsynonymous SmV or nonaltered SMC5/6 genes.

### Survival analysis

Overall survival time and status of patients included in TCGA and PCAWG were obtained from cBioPortal (see the ‘Data availability’ section). Overall survival time for patients from the Hartwig dataset was counted from the day they joined the study to the date of their death or last follow up date. Only patient response to treatment admitted after biopsy was accessed in this study. Constructing and analyzing Kaplan–Meier curves was handled using R packages ggsurvfit (version 1.1.0), survminer (version 0.4.9), and survival (version 3.8.3).

### Statistical analysis

All statistical tests were performed in R with ggpubr (version 0.6.0). Pairwise comparison for TMB, aneuploidy, and ploidy score was assessed by Wilcoxon rank-sum test. Constructing and analyzing Kaplan–Meier curves was handled using R packages ggsurvfit (version 1.1.0), survminer (version 0.4.9), and survival (version 3.8.3). Pairwise log-rank test was used for survival analysis.

### Ethics statement

This study involved secondary analysis of existing, de-identified human genomic datasets and did not constitute new human subjects research. Hartwig Medical Foundation data were obtained under a signed Data Use Agreement (reference: DR-435). All data were used in accordance with the terms of the respective access agreements, and no attempt was made to re-identify participants. As this study used only pre-existing, de-identified data obtained through established institutional access mechanisms, independent ethical review by an Institutional Review Board was not required under institutional policy. Informed consent was obtained from all participants by the originating studies prior to data collection. We acknowledge the patients and families who contributed samples to TCGA, PCAWG, and the Hartwig Medical Foundation, without whom this research would not be possible.

## Results

### SMC5/6 variants occur across human tumors

**Figure 1.**
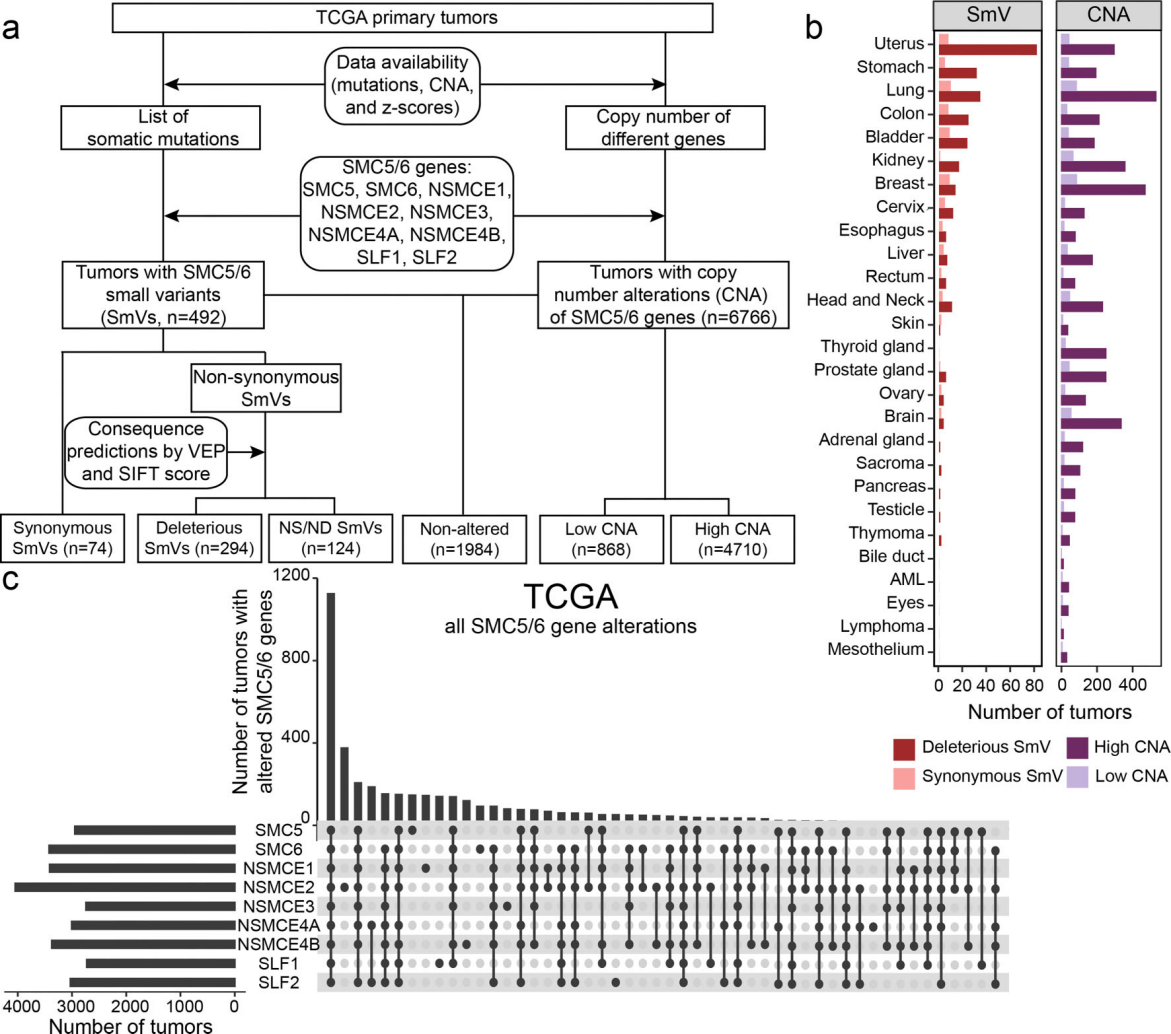
Alterations in SMC5/6 complex genes are evident throughout human cancers in TCGA. (**A**) Flow chart depicting the workflow to assess SMC5/6 gene alterations in tumor exome sequences from TCGA. Exome sequences were filtered as shown. SmVs include base substitutions and indels <50 bp. Deleterious SmV were classified based on genetic (nonsynonymous), evolutionary conservation (VEP), and biochemical (SIFT score) prediction methods. NS/ND SmV represent nonsynonymous variants that were not predicted to be deleterious. Tumors with both SmV and CNA were included in both cohorts. Tumors with both high and low CNA of different SMC5/6 genes were excluded from all CNA cohorts. Tumors with parallel CNA (all high or all low) of multiple SMC5/6 genes were included. (**B**) Number of tumors with SMC5/6 gene alterations classified by tumor/tissue type. (**C**) UpSet plot depicting frequency of alterations (SmV and CNA) in SMC5/6 genes. Left bar graph indicates number of tumors with any alteration in indicated SMC5/6 genes. Top bar graph indicates number of tumors with combinations of SMC5/6 gene alterations.

To assess whether SMC5/6 genes are altered in human cancer, we first analyzed all primary tumor exome sequences within TCGA. We investigated nine genes: those encoding the six subunits of the core complex (SMC5, SMC6, NSMCE1–4), including the two genes encoding NSMCE4 (NSMCE4A and NSMCE4B), and the SLF1 and SLF2 subunits (Fig. 1A). In a previous study of SMC5/6 variants in cancer, all SMC5/6 gene aberrations were assessed in aggregate [29]. To discern whether certain types of SMC5/6 gene aberrations resulted in different outcomes, we narrowed the cohorts evaluated into two separate groups of human tumors: those with somatic mutations (SmV including base substitutions and insertions/deletions) in SMC5/6 genes, and those with CNAs in SMC5/6 genes. The tumors with SmV in SMC5/6 genes were further divided into two subgroups: those predicted to be deleterious (i.e. frame shift, missense) [31, 32] versus those predicted to be benign (synonymous or nonsynonymous nondeleterious (NS/ND) SmV). SMC5/6 copy number changes were categorized as low (<2) or high (>2) subgroups. Exomes containing neither SmV nor CNA in all SMC5/6 genes made up the nonaltered group.

Nearly three-quarters of tumors in TCGA displayed CNA in SMC5/6 genes (*n* = 6766; of which 1188 were excluded from further analysis for having both low and high CNA of different SMC5/6 genes), while 1984 tumors retained wild-type SMC5/6 genes with a normal copy number. A total of 492 tumors exhibited SmV in at least one SMC5/6 complex gene, most of which harbored SmVs that were predicted to be deleterious (Fig. 1A). While SMC5/6 CNA was a relatively common occurrence across different tumor types, tumors exhibiting the most SMC5/6 gene SmVs originated in uterus, gastrointestinal tract, and lung (Fig. 1B). The majority of tumors with alterations in >1 SMC5/6 genes exhibited SmV and/or CNA of all nine genes (*n* = 1128, Fig. 1C), most of which was due to copy number changes likely reflecting global aneuploidy of a tumor. Tumors in which only one SMC5/6 gene was altered ranged from *n* = 21–371 (Fig. 1C). Among tumors with only a single SMC5/6 gene altered, *NSMCE2* was most frequently altered when assessing all possible gene variants (Fig. 1C), but *SMC6* most frequently incurred SmV (Supplementary Fig. S1A).

When SmV were assessed independently, the majority were found in *SMC5* and *SMC6* which comprise the largest open reading frames of all nine genes (Supplementary Fig. S1A). In contrast to the analysis of all variants including copy number changes (Fig. 1C), a minority of tumors exhibited SmV in more than one SMC5/6 complex gene (Supplementary Fig. S1A). Prior studies have demonstrated that loss of a single SMC5/6 complex component led to disruption of the entire complex and depletion of additional components [7, 9, 39]; thus, the lack of overlapping gene SmVs in tumors may be due to the fact that disruption of a single SMC5/6 gene by SmV is sufficient to interfere with function of the entire complex. To determine whether deleterious SmV occurred in a specific subunit, we quantified the number of tumors with SmV in each SMC5/6 gene. Tumors with SmV in *SMC5* and *SMC6* genes represented the largest group (Supplementary Fig. S1B), likely because they are the largest genes in the complex. When controlling for gene size, there did not appear to be a specific subunit that incurred more deleterious SmV (Supplementary Fig. S1B). Additionally, allele frequency of SmV was similar for all genes and indicated a pattern of allelically imbalanced SMC5/6 gene alteration (Supplementary Fig. S1C).

### Recurrent SMC5/6 SmV in cancer are rare

**Figure 2.**
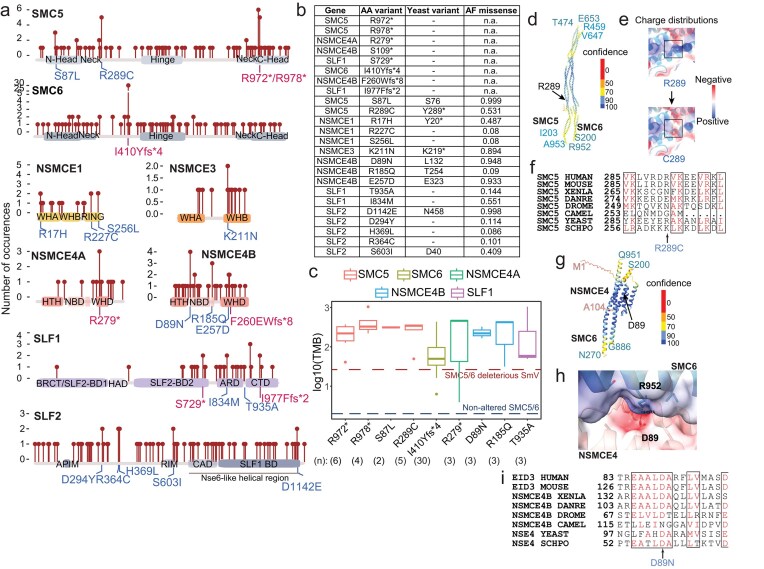
SMC5/6 dysfunction in cancer is not caused by hotspot mutations. (**A**) Lollipop chart showing location and frequency of deleterious SmV detected in TCGA tumor exomes within SMC5/6 complex genes. Each SMC5/6 subunit is shown with its known conserved structural and functional domains annotated. Two types of mutations are highlighted: truncating mutations (red) and single amino acid substitutions (blue). WH, winged helix; BRCT, BRCA1 C-terminal; BD, binding domain; IDR, intrinsic disordered region; ARD, Ankyrin repeat domain; CTD, C-terminal domain. (**B**) Table depicts most frequently occurring mutations for each SMC5/6 gene including amino acid (AA) change, yeast homology site, and AlphaFold missense pathogenicity score. Homologous sites in yeast are not listed for truncation mutants or poorly conserved/unstructured regions. (**C**) Box and whisker plot shows TMB in exomes containing most frequently occurring SMC5/6 gene SmV. Red dotted line shows average TMB for exomes with deleterious SMC5/6 SmV, blue dotted line shows average TMB for exomes with no SMC5/6 gene alteration. Number in parenthesis indicates the number of times each mutation was detected in independent exomes. (**D**) AlphaFold-predicted model of the human SMC5/6 arm region (ipTM = 0.52, pTM = 0.52), colored by model confidence (pLDDT). The residue ranges used for prediction are indicated in the corresponding subunit colors, and the mutated site is marked in black. (**E**) Close-up view of residue R289 in SMC5 with substitution of arginine (R) to cysteine (C). (**F**) Sequence alignment of the SMC5 arm region across representative species. (**G**) AlphaFold-predicted model of the human EID3 HTH domain and its interface with part of SMC6 arm (ipTM = 0.83, pTM = 0.78). The predicted model is colored by pLDDT. The residue range used for prediction is indicated in subunits’ corresponding colors, and the mutated site is indicated in black. (**H**) A close-up view of D89 with electrostatic surface and alignment of NSMCE4 D89 residue in proximity to SMC6-R952. (**I**) Sequence alignment of NSMCE4 (EID) D89 across representative species.

SMC5/6 is a multifunctional complex with both structural and enzymatic functions. Enzymatic functions can be mapped to specific domains of individual subunits and structural functions are enacted by multisubunit interactions [2, 3, 40, 41]. For example, structural studies of the yeast Smc5/6 complex demonstrated that double-strand DNA binding is enacted through the heads domains of Smc5 and Smc6, as well as the Nse3 (NSMCE3) and Nse4 (NSMCE4) subunits [40, 42]. We mapped the deleterious SmVs of SMC5/6 found in human tumors in TCGA to assess whether a specific domain was frequently mutated. Domain annotations were obtained by integrating human SMC5/6 subunit domain mapping and the yeast Smc5/6 subunit structures mapped by cryo-EM studies [40, 43, 44]. We found that SMC5/6 deleterious SmV occurred throughout structural and functional domains (Fig. 2A). Despite identifying 363 unique deleterious SmV in SMC5/6 genes across 492 human tumors, we found very few recurrent mutations (Fig. 2A). We assessed the SmV that occurred most frequently in each SMC5/6 gene and found approximately two-thirds were missense mutations (Fig. 2B). When these missense variants were analyzed by AlphaFold only 50% had a pathogenicity score >0.5 (Fig. 2B), suggesting that some missense SmV do not result in detrimental changes to protein. To understand the pathogenic effects of frequent SmVs, we assessed both associated TMB and homology to yeast Smc5/6 structure. We found that the mutational burden within tumors with the most frequently occurring SmV was substantially higher than the average TMB for all tumors with SMC5/6 deleterious SmV (Fig. 2C), suggesting that disruption of SMC5/6 through frequently occurring variants is associated with higher levels of tumor mutagenesis.

As no cryo-EM map of the human SMC5/6 complex is available, we relied on the structure of the homologous yeast Smc5/6 complex [40, 43, 44] and AlphaFold-predicted structures to assess the functional impact of SMC5/6 variants found in cancer. While not all tumor-associated SMC5/6 SmV had homologous sites in yeast (Fig. 2B), two recurrent missense mutations, SMC5 R289C and NSMCE4B D89N, were mapped to homologous regions and subjected to homology modeling. R289C lies in the coiled-coil domain of the SMC5 protein (Fig. 2D) and is predicted to reduce local positive charge. The R > C change at position 289 may disrupt electrostatic interactions along the coiled-coil interface between SMC5 and SMC6, potentially affecting stability or intrasubunit contacts (Fig. 2E). Sequence alignment of the SMC5 arm region across species shows limited conservation at position 289 (Fig. 2F), suggesting that the residue may contribute to fine-tuning rather than an essential conserved function. NSMCE4B D89 lies in the HTH domain of the protein and its interface with part of the SMC6 arm region (Fig. 2G). NSMCE4B D89 is relatively well conserved and is predicted to interact with SMC6 R852 (Fig. 2H and I), an interaction that is also seen in the yeast Smc5/6 structure [40]. These predictions suggest that cancer-associated deleterious SMC5/6 variants have potential to impact integrity and function of the complex.

When mapped to lengthwise renderings of SMC5/6 genes, the pattern of SmV was dispersed across all coding regions with few recurrent mutations (Fig. 2A). This “long and low” pattern of variants is in striking contrast to that of well-studied oncogenes and tumor suppressors such as TP53 and PIK3CA which exhibit specific “hotspot” cancer-associated mutations (Supplementary Fig. S2). Interestingly, this long and low pattern of SmV was also observed for cohesin gene mutations in cancer (Supplementary Fig. S3). Cohesin is an SMC protein complex that, in contrast to SMC5/6, has an established role in tumorigenesis [45]. Given the essential interactions between many genes and domains of a protein complex, a long and low pattern of variants may be equally disruptive as hotspot mutations in individual oncogenes.

### SMC5/6 variants are associated with increased TMB

**Figure 3.**
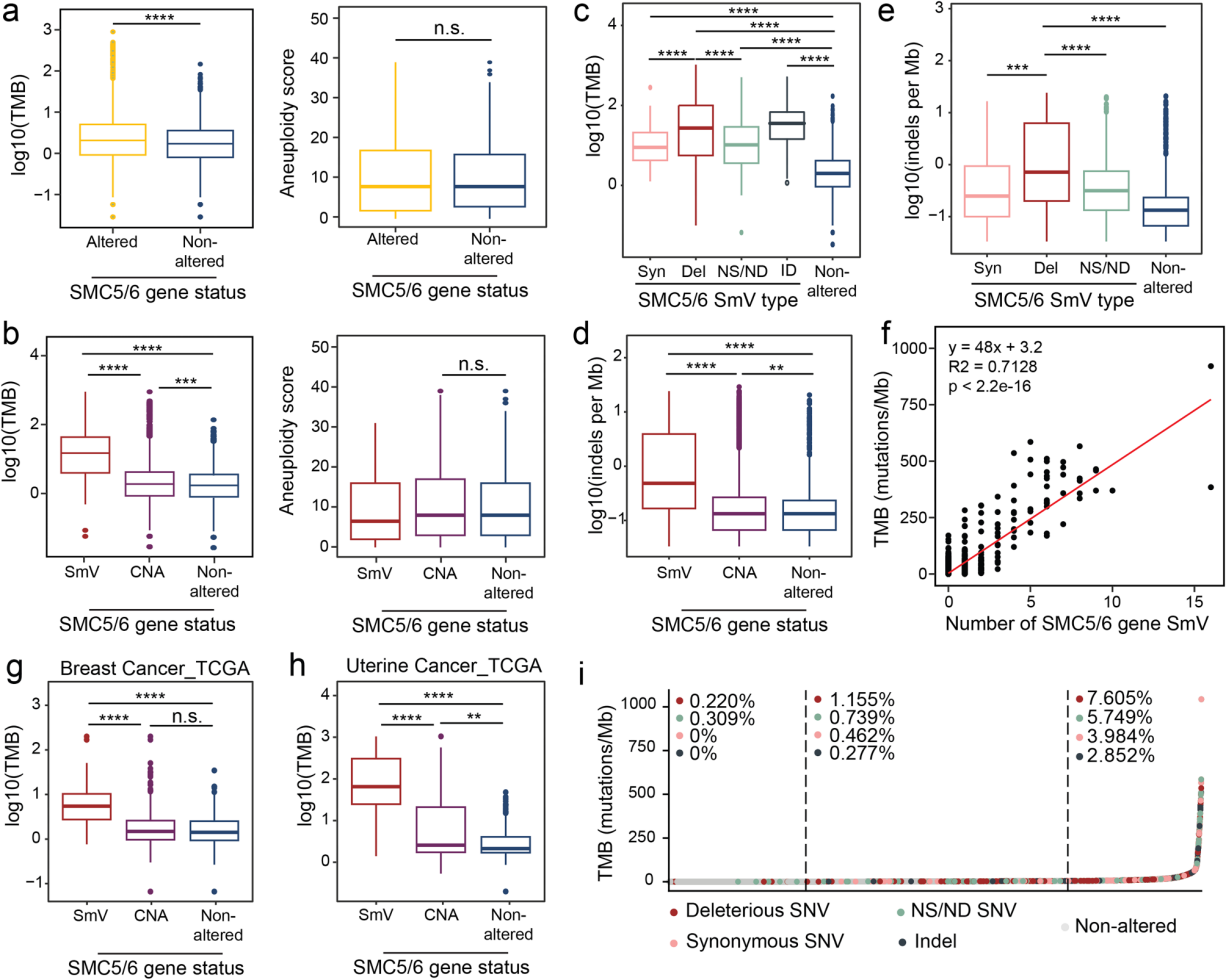
SMC5/6 dysfunction correlates with elevated TMB in TCGA exomes. (**A, B**) TMB and aneuploidy score of TCGA exomes (**A**) with or without any alteration (SmV or CNA), or (**B**) divided further into SmV, CNA, or nonaltered SMC5/6 genes. (**C**) TMB of TCGA exomes categorized by type of SMC5/6 SmV including deleterious, synonymous, and NS/ND single nucleotide variants (SNVs) and small indels (ID). Significance determined by Wilcoxon rank-sum test. (**D, E**) Quantitation of genome-wide indels in tumor exomes grouped by SMC5/6 gene status as indicated. Significance determined by Wilcoxon rank-sum test. (**F**) Dot plot depicting correlation between TMB and number of SMC5/6 gene SmV per exome. A linear best-fitting regression is shown in red. The corresponding linear equation, Pearson correlation coefficient (R^2^), and *P*-value are provided. (**G, H**) TMB of TCGA exomes from patients with breast (**G**) or uterine (**H**) cancer divided into genomes with SmV, CNA, or nonaltered SMC5/6 genes. Significance determined by Wilcoxon rank-sum test. (**I**) TMB of each exome in TCGA is plotted from lowest to highest. Color designates type of SMC5/6 SmV in each exome. Dotted lines denote the lower and upper quartiles. Frequency of each type of SmV per section is shown. X^2^ test for independence was 885.3, df = 8, *P *< 2.2e-16. **P <.01, ***P <.001, ^****^P <.0001.

To assess the functional impact of somatically acquired SMC5/6 variants, we evaluated genome instability in cancers with altered SMC5/6 genes in TCGA. When tumors with SMC5/6 SmV and CNA were pooled, we found an increase in TMB relative to tumors with nonaltered SMC5/6 genes (Fig. 3A). Interestingly, we found no difference in aneuploidy between these groups (Fig. 3A), differing from a recently reported association between SMC5/6 gene alterations and aneuploid breast tumor genomes [29]. Based on these data, we asked whether SMC5/6 SmV and CNA similarly affected TMB. An increase in TMB was found in the pool of tumors with SMC5/6 gene CNA relative to nonaltered (median 2.2 and 2 mutations/Mb, respectively); however, a striking increase in TMB was noted in tumors with SMC5/6 SmV (17.3 mutations/Mb) relative to tumors with either SMC5/6 CNA or nonaltered SMC5/6 (Fig. 3B). When CNAs in SMC5/6 genes were further narrowed to groups of low (<2 copies) or high (>2 copies), we found a statistically significant difference in TMB distribution but similar median levels of TMB when compared to tumors with nonaltered SMC5/6 genes (range 2–2.3 mutations/Mb, Supplementary Fig. S4A and B). When comparing tumors grouped by SmV, CNA, or nonaltered SMC5/6 genes, we again found no alteration in aneuploidy score (Fig. 3B and Supplementary Fig. S4C).

To complement the analysis of TCGA exomes, we analyzed tumor whole genomes from PCAWG and found 1923 tumors with SMC5/6 gene alterations (Supplementary Fig. S5A). Similar to tumors in TCGA, those in PCAWG with SMC5/6 SmV displayed elevated TMB relative to tumors with nonaltered SMC5/6 genes (Supplementary Fig. S5B). While PCAWG tumors with SMC5/6 CNA had an elevated TMB relative to those with nonaltered SMC5/6, the level remained significantly lower than tumors with SMC5/6 SmV (Supplementary Fig. S5B). Ploidy score was significantly different in tumors with SMC5/6 gene alterations relative to nonaltered, but median values were relatively similar among the cohorts and close to diploid (median values range 1.94–2.08 chromosome sets per cell), suggesting that statistical differences were driven by outliers (Supplementary Fig. S5C). These data indicate that SMC5/6 disruption, and specifically SmV, are associated with increased TMB.

When assessed independently, variants in all SMC5/6 genes were associated with elevated TMB in TCGA exomes (Supplementary Fig. S6A). Similarly, CNA of each SMC5/6 gene assessed independently did not reveal any association with altered TMB (Supplementary Fig. S6B). However, when we categorized tumors by the type of SmV—deleterious, synonymous, or nonsynonymous/nondeleterious SNV, or indel—we found that the SmV predicted to have most disruptive impact on SMC5/6 gene products (deleterious SNV and indel) were associated with the highest TMB (Fig. 3C). We independently assessed indel burden in tumor genomes and found that indel burden was also elevated in tumors with altered SMC5/6 genes, and more so in tumors with deleterious mutations in SMC5/6 genes (Fig. 3D and E). Further, a striking correlation between number of SMC5/6 SmV within a tumor and mutational burden was observed (Fig. 3F). These data suggest that the degree of SMC5/6 dysfunction incurred by a tumor may impact mutational burden.

As prior data demonstrated that SMC5/6 gene alterations in breast cancer correlated with increased genome instability, we investigated disease-specific cohorts. We examined TMB in breast, lung, and uterine cancer genomes as all cohorts had a substantial number of SMC5/6 gene alterations (both SmV and CNA). Across all disease-specific cohorts we saw that genomes with SMC5/6 gene SmV had a significantly higher TMB than those with nonaltered or copy number altered SmV (Fig. 3G and H, and Supplementary Fig. 7A). Similar to our pan-cancer analysis, TMB was elevated in breast, uterine, and lung tumors with deleterious SMC5/6 SmV in comparison to those with nondeleterious variants (Supplementary Fig. 7B–D). Disease-specific cohorts did not exhibit altered TMB in tumors with high compared to low SMC5/6 CNAs (Supplementary Fig. 7B–D). Disease-specific cohorts also did not exhibit elevated aneuploidy in tumors with SMC5/6 alterations, consistent with our pan-cancer analysis (Supplementary Fig. 7E–G). These data demonstrate that the predominant marker of genome instability associated with SMC5/6 gene disruption is TMB and that this applies to both pan-cancer and disease-specific groups.

### Elevated TMB and SMC5/6 SmV: the chicken or the egg?

The correlation between SMC5/6 SmV and high TMB can be explained by two models: the first model suggests that dysfunction of SMC5/6 caused by SmV may enable an increase in TMB, while the second model posits that tumors with a high mutational rate could incidentally result in SmV of SMC5/6 genes. The second model, therefore, predicts an equal distribution of SmV types across each SMC5/6 gene. Instead, we found that the majority of SmV were predicted to be deleterious in nearly all SMC5/6 genes (Supplementary Fig. S1B). The second model also predicts a similarly elevated TMB across all SmV types. In contrast, we found that deleterious SNV and indels, which are more likely to result in dysfunction of SMC5/6 gene products, are associated with significantly higher TMB than synonymous or nonsynonymous/nondeleterious (NS/ND) variants (Fig. 3C). In this analysis, we sorted TCGA exomes from lowest to highest TMB, divided the cohort into quartiles, and assessed the occurrence of SMC5/6 SmV in each quartile (Fig. 3G). The second model predicts that the type of SmV would occur with similar frequency across each quartile. While deleterious, synonymous, and NS/ND SNV were all enriched in highest quartile (Fig. 3I), the fraction of tumors with deleterious SNV was significantly higher than those with synonymous or NS/ND SNV [by Chi-squared test, *X^2^* (8, *n* = 8809) = 885.3, *P* < 2.2e-16]. We further analyzed SmV by separating indels from SNV. Indels were a less frequent mutation type than SNV among SMC5/6 genes but were also associated with elevated TMB and were enriched in the highest TMB quartile (Fig. 3I). As indels are predicted to result in the most deleterious outcomes for SMC5/6 gene products, we independently analyzed tumors with SMC5/6 indels. To account for the potential of an indel-causing mutagenic process that incidentally causes SMC5/6 gene indels, we measured only SNV burden in the genomes of tumors with SMC5/6 gene indels. We found that the tumors with highest SNV harbored the highest frequency of SMC5/6 gene indels (Supplementary Fig. 8). While we cannot conclusively establish which model explains the relationship between SMC5/6 dysfunction and TMB, these data support the first model of directional correlation in which dysfunction of SMC5/6 drives TMB.

### SMC5/6 disruption predicts response to immunotherapy

**Figure 4.**
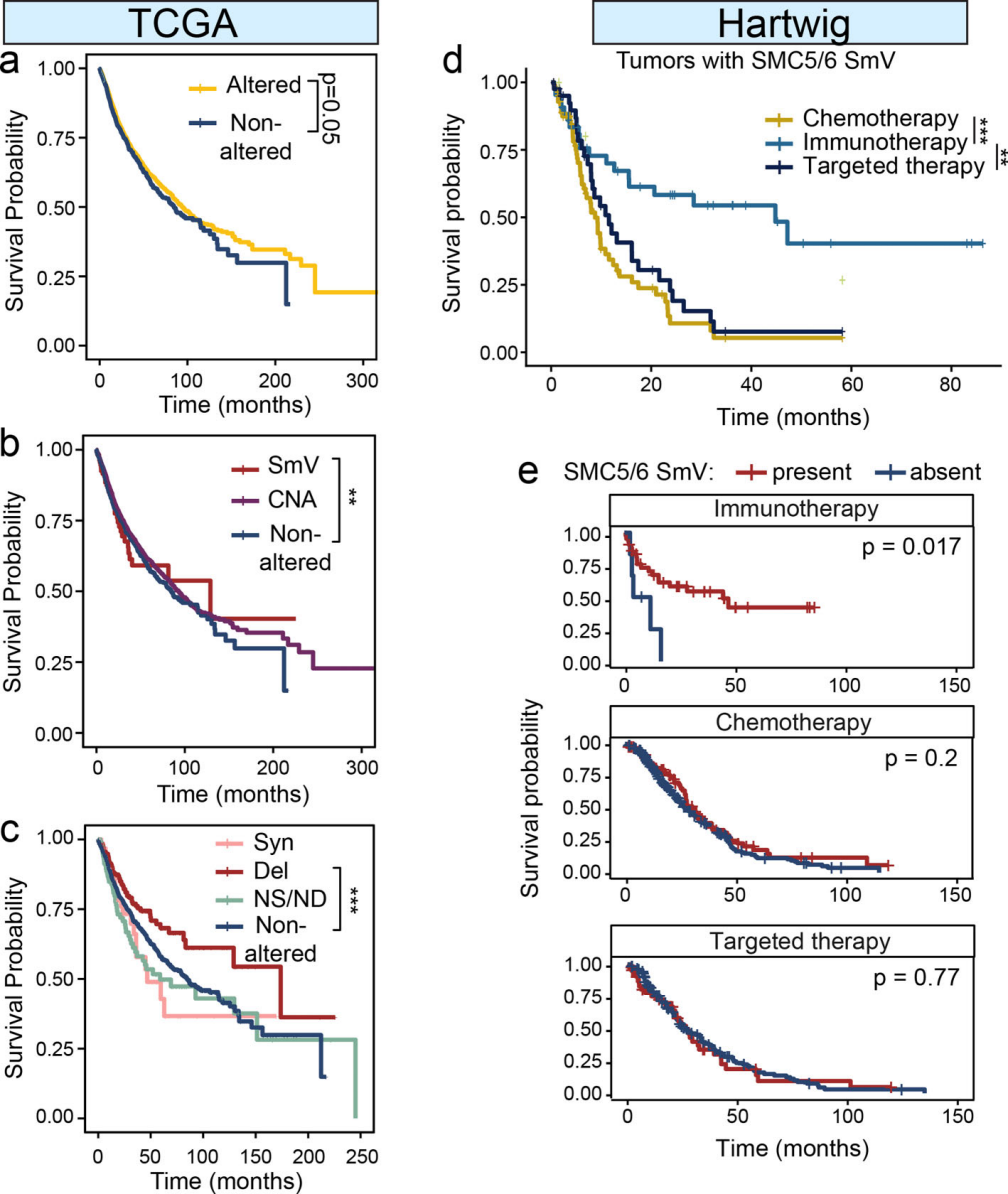
SMC5/6 disruption is correlated with improved patient survival. (**A–C**) Kaplan–Meier curves show survival of patients in TCGA cohort with (**A**) any SMC5/6 gene alteration or nonaltered SMC5/6 genes, (**B**) SmV, CNA, or nonaltered SMC5/6 genes, (**C**) categorized by type of SMC5/6 SmV. (**D–E**) Kaplan–Meier curves show survival of patients in Hartwig colorectal cancer cohort separated by therapy received. (**D**) All patients with SMC5/6 SmV detected in tumor genomes are shown according to treatment type. (**E**) Recipients of specific cancer therapies are stratified by SMC5/6 SmV status (present versus absent). *P*-values determined by log-rank test, **P <.01, ***P <.001.

To assess the phenotypic effect of SMC5/6 gene variants in cancer, we evaluated overall survival of patients with nonaltered or altered SMC5/6. From tumor genomes in TCGA, we found that any alteration in SMC5/6 was associated with improved survival (Fig. 4A). Tumors with SMC5/6 gene SmV had improved survival over those with no SMC5/6 alterations (Fig. 4B), and deleterious SMC5/6 SmV predicted the best survival among all tumors stratified by SMC5/6 SmV status (Fig. 4C). Tumors with very high TMB, termed “hypermutator” tumors, are more susceptible to immune clearance, and are therefore more sensitive to immunotherapeutic approaches [46, 47]. We hypothesized that the improvement in survival of patients with tumors harboring SMC5/6 gene SmV may be due to susceptibility to immunotherapy. To test this possibility, we interrogated a dataset of metastatic colorectal cancer from the Hartwig Medical Foundation with thorough annotation of treatment and survival [30]. After filtering, 545 tumor genomes with available treatment and survival data were included in our analysis. A subgroup analysis of colorectal tumors with SMC5/6 SmV demonstrated improved survival in patients treated with immunotherapy relative to other treatment groups (Fig. 4D). We then assessed patient survival of patients with and without SMC5/6 gene SmV following specific therapies. We found that patients with SMC5/6 SmV demonstrated significantly improved survival upon treatment with immunotherapy, and this significant increase in survival was specific to immunotherapy (Fig. 4E). From these findings across two large databases of cancer, we conclude that SMC5/6 SmV improves responses to immunotherapy, likely by promoting increased TMB.

### SMC5/6 disruption enables mutagenesis by POLE defects and MMRd

**Figure 5.**
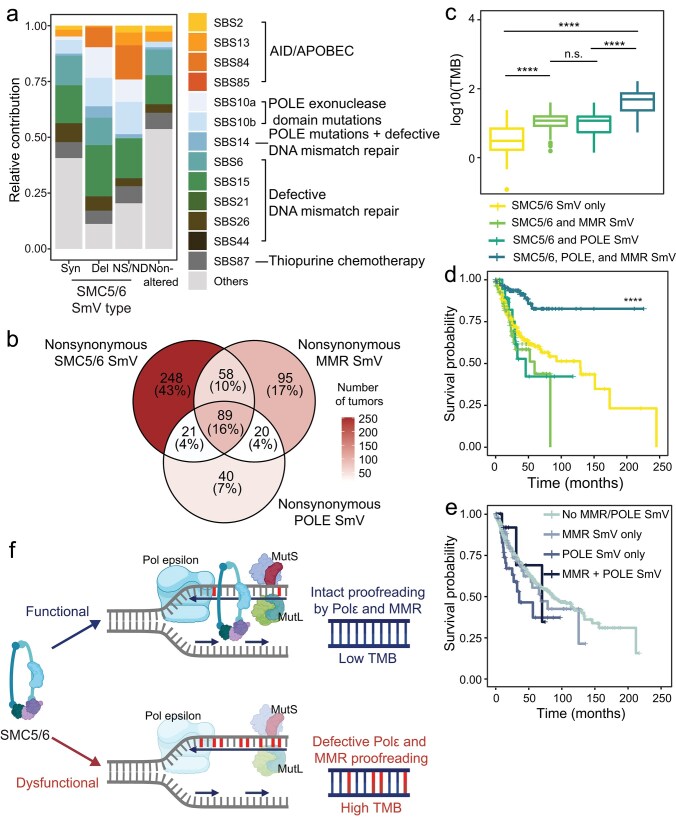
Increased TMB in SMC5/6-variant tumors reflects POLE dysfunction and MMR deficiency. (**A**) Relative contribution of SBS signatures from TCGA exomes categorized by SMC5/6 SmV type. Colored signatures are designated with etiology in legend; these signatures comprised >5% relative contribution of TMB in SMC5/6 deleterious SmV cohort. Signatures in gray are designated “others” and comprised <5% relative contribution of TMB in deleterious SmV cohort. (**B**) Venn diagram shows confluence of TCGA tumors with nonsynonymous SmV in SMC5/6 genes, MMR genes, and POLE. Color gradient shows number of tumors in each group. (**C**) TMB and (**D**) Kaplan–Meier curve of TCGA exomes with nonsynonymous SmV in SMC5/6 genes, MMR genes, and/or POLE. Significance determined by Wilcoxon rank-sum test ^****^P <.0001, pairwise comparisons between the cohort with SmV in all (SMC5/6, MMR, POLE) relative to each other group is shown. (**E**) Kaplan–Meier curve of TCGA exomes with nonsynonymous SmV in MMR genes, and/or POLE but nonaltered SMC5/6 genes. All pairwise comparisons are not statistically significant. (**F**) Model depicting potential effect of SMC5/6 in supporting proofreading by POLE and MMR on the leading strand. Through either structural or catalytic functions, SMC5/6 enables proofreading capacity of POLE and/or MMR. In the absence or dysfunction of SMC5/6, which may occur through variants detected in cancer genomes, proofreading fails and mutagenesis ensues.

To define the mutational processes that drive elevated TMB in tumors with SMC5/6 variants, we pooled TCGA exomes based on SMC5/6 SmV type and assessed for SBS mutational signatures [48–50]. Compared to tumor exomes with nonaltered SMC5/6, tumor exomes with SMC5/6 SmV were enriched for SBS and indel signatures attributed to POLE dysfunction and defective MMR (MMRd) indicating that these mutational processes are correlated with SMC5/6 dysfunction (Fig. 5A and Supplementary Fig. 9A). MMRd and POLE mutational processes are well-established drivers of the “hypermutator phenotype” in colorectal, endometrial, and other cancers [51–54]. To determine the effect of SMC5/6 disruption in combination with these mutagenic processes, we defined subgroups of TCGA tumors based on all nonsynonymous variants in SMC5/6 genes, MMR genes, or POLE genes, and combinations of these three subgroups (Fig. 5B). As expected, the subgroups were enriched for SBS signatures defining each deficiency: MMR variant tumors were enriched for MMRd signatures and POLE variant tumors had elevated POLE-associated SBS signatures (Supplementary Fig. S9B). We found that SMC5/6 variants synergize with MMRd and POLE dysfunction, leading to elevated mutational burden and improved survival relative to tumors with only SMC5/6 variants (Fig. 5C and D). Tumors with multiple variants in SMC5/6, MMR, and POLE genes displayed improved survival over those without variants or with variants in only one category (Supplementary Fig. S9C and D). The combination of all three (SMC5/6, MMR, and POLE variants) resulted in the highest TMB and best survival (Fig. 5C and D). Interestingly, among TCGA exomes with nonaltered SMC5/6 genes, nonsynonymous SmV in MMR genes and POLE did not result in better patient outcomes (Fig. 5E). These data suggest that disruption of the SMC5/6 complex in cancer exacerbates mutagenesis caused by POLE defects and MMRd, and may serve as a biomarker for response to immune checkpoint blockade.

## Discussion

The SMC5/6 complex has multiple functions in genome maintenance and germline mutations in SMC5/6 genes are detrimental to the development and health of humans and model organisms. However, little is known about the consequences of somatic SMC5/6 dysfunction in cancer. In this pan-cancer study, we show that disruption of SMC5/6 genes correlates with high TMB. Similarly elevated TMB is found in other tumors with DNA repair defects [55, 56], highlighting the importance of the SMC5/6 complex in genome stability. In principle, genome plasticity associated with SMC5/6 dysfunction could result in cancer cell evolution and heterogeneity, leading to poor patient outcomes [57]. Indeed, this was suggested in a study of SMC5/6 gene alterations in breast cancer [29]. In contrast, our data demonstrate that, across all cancers, SmV in SMC5/6 are associated with improved survival (Fig. 4A–C). In a dataset of several hundred patients with metastatic colorectal cancer, SMC5/6 SmV correlated specifically with improved responses to immunotherapy (Fig. 4D and E). These findings are analogous to the reported responses to immunotherapy in hypermutated tumors with MMRd and POLE dysfunction [46, 47]. Interestingly, we find that somatic disruption of SMC5/6 is correlated with mutagenesis from MMRd and POLE dysfunction. Additionally, SMC5/6 gene variants in cancer augment mutational burden in tumors with MMRd or POLE mutations, and predict superior patient survival relative to MMRd or POLE mutations alone (Fig. 5). These data indicate that somatic alteration of SMC5/6 function in cancer may enable hypermutator phenotypes caused by MMRd and POLE dysfunction. We propose a model in which SMC5/6 functions to support proofreading by POLE and MMR directly or indirectly to limit mutagenesis during DNA synthesis (Fig. 5F). This SMC5/6 function may be relevant during normal DNA replication but under conditions of replicative stress, such as in cancer, SMC5/6 dysfunction may result in higher rates of mutagenesis. When coupled with dysfunction of either POLE or MMR, the mutagenic rate increases (as in Fig. 5C).

Genetic and biochemical interactions between Smc5/6 and POLE were established in yeast studies in which Smc5/6 was found to directly interact with and sumoylate the POLE Pol2 subunit [58, 59]. Sumoylation of the yeast Pol2 protein, which occurred during S phase and upon replication stress, was necessary for replication progression and prevention of recombination-mediated chromosomal rearrangements [58, 59]. In cancer, replication stress is ubiquitous, thus replicative polymerases and replication stress responses are essential to maintain high rates of DNA synthesis, enable genome duplication, and promote cell and tumor growth [60, 61]. Though the presence and function of sumoylated POLE in mammalian cells has not been confirmed, it is conceivable that dysfunction of SMC5/6 in cancer could limit POLE sumoylation leading to decreased polymerase function and/or fidelity and an increased mutational rate. This model would explain the finding of POLE dysfunction mutational signatures in tumors with deleterious variants in SMC5/6. No correlation between SMC5/6 and MMR has been reported, though our data suggest that SMC5/6 supports MMR, a prediction warranting future biological studies.

The specific function or domain of the multifunctional SMC5/6 complex that supports high-fidelity DNA synthesis by POLE and DNA repair by MMR is unknown since no hotspot SmV in SMC5/6 were found in our study, similar to a previous report [29]. The “long and low” pattern of SMC5/6 SmV was also noted in cohesin genes in our study and in others in which expanded cohorts were analyzed [45], but in stark contrast to most tumor suppressor and oncogene SmV. Thus, we postulate that somatic dysfunction of protein complexes can be achieved through disruption of numerous genetic loci rather than a single point of inactivation or hyperactivation. This is supported by homology modeling of SMC5/6 cancer variants using the yeast Smc5/6 structure which predicted subtle changes to functional domains and protein subunit interactions. Most cancer-associated SMC5/6 SmV were hetero/hemizygous, which is expected given that SMC5/6 is essential for cell proliferation. We found a cohort of tumors that harbored more than one variant in SMC5/6 genes and were associated with particularly elevated TMB suggesting the possibility that combinations of allelically imbalanced SMC5/6 SmV cause more substantial complex dysfunction. Our *in silico* study did not evaluate the specific biological function of each SMC5/6 SmV (or combinations of SmV), but the correlation between somatically acquired deleterious SMC5/6 variants and increased TMB demonstrates a previously undefined role for SMC5/6 in preventing base substitutions.

Our study lays a foundation for further investigation into the clinical implications of SMC5/6 dysfunction in cancer. Our analysis was limited by the assumption that SMC5/6 variants identified in cancer genomes were somatically acquired, although it is possible that they represent heterozygous germline variants. While patients with biallelic germline SMC5/6 variants have extreme developmental phenotypes and die early in life, their parents with heterozygous germline variants have not been reported to be cancer predisposed. However, the sample size is small to date and future investigation into whether heterozygous germline SMC5/6 variants predispose to mutagenesis and malignancy is warranted. SMC5/6 variants correlate with a survival benefit in patients treated with immunotherapy and in cases of combined SMC5/6 variants with POLE mutation or MMRd. These findings suggest that SMC5/6 genes should be included in diagnostic targeted sequencing panels to enable prognostic predictions and tailored therapeutic approaches.

## Supplementary Material

zcag012_Supplemental_File

## Data Availability

Somatic variant calls and copy number information of the TCGA projects were obtained using R package TCGA biolinks (version 2.32.0), while whole genome sequencing data of the International Cancer Genome Consortium (ICGC) was downloaded at https://dcc.icgc.org/releases/PCAWG (release 28). Clinical information, aneuploidy and ploidy scores for both TCGA and PCAWG (ICGC) dataset were obtained from cBioPortal on 15 January 2024. Sample and patient identification numbers were utilized to match data across these databases. Somatic variant calling and clinical data of colorectal metastases were performed and reported by the Hartwig Medical Foundation (study number NCT01855477). All original code used for data presented is available at https://doi.org/10.5281/zenodo.19684599.
