# Supplementary material for: Disruption of the structural maintenance of chromosomes 5/6 complex enables tumor mutagenesis
Source: NAR Cancer. 2026 May 21;8(2):zcag012. doi: 10.1093/narcan/zcag012 (PMC13191284; doi:10.1093/narcan/zcag012)
Supplement: zcag012_Supplemental_File [file zcag012_supplemental_file.pdf]

SUPPLEMENTARY DATA

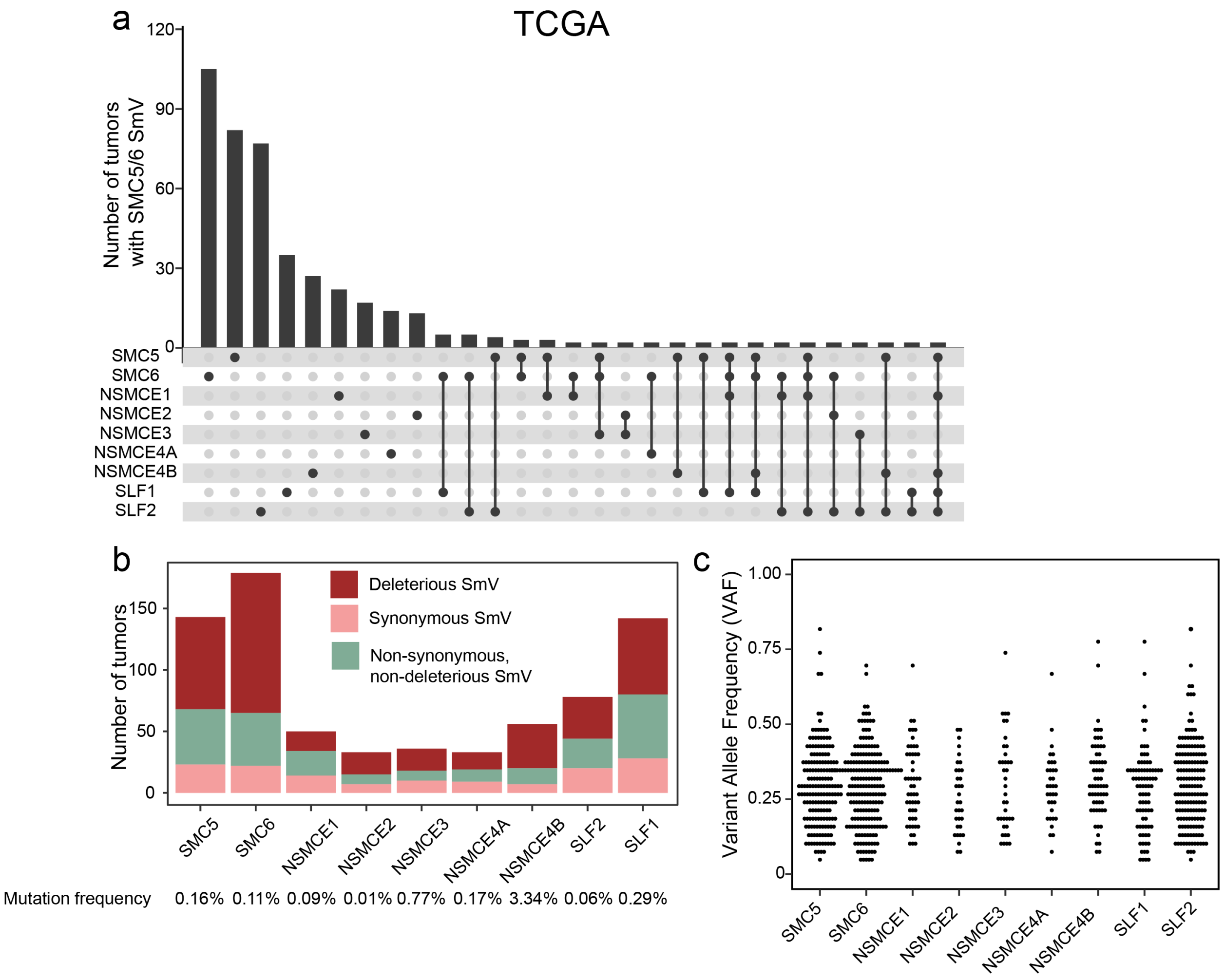

Supplemental Figure 1. Tumor-associated SmV occur across all SMC5/6 complex genes. (**a**) UpSet plot depicting frequency of SMC5/6 SmV in tumors from TCGA. (**b**) Type and number of SmV in each SMC5/6 gene found in TCGA exomes. Mutation frequency reflects the number of unique SmV relative to size of each gene. (**c**) Variant allele frequency (VAF) of SMC5/6 SmV in TCGA exomes. Each dot represents VAF of the indicated gene from an individual tumor. Only tumors with SMC5/6 SmV are included.

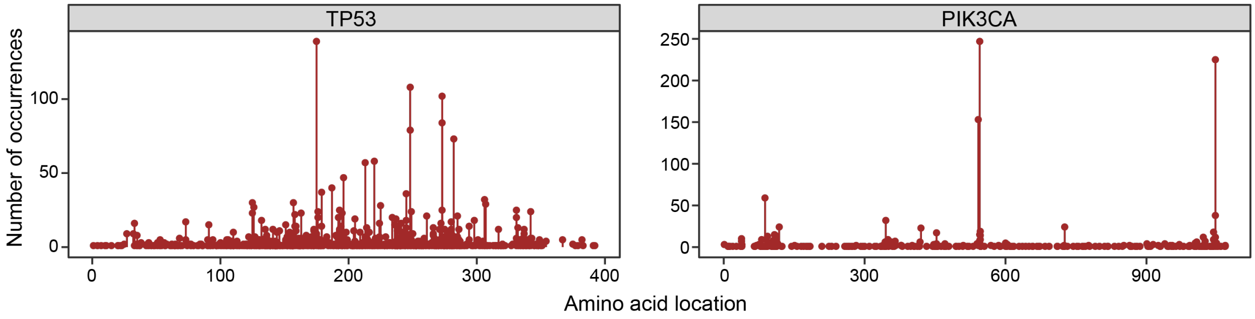

Supplemental Figure 2. SmV in tumor suppressors and oncogenes occur at hotspots. Lollipop charts depicting frequency of deleterious TP53 and PIK3CA SmV identified in TCGA.

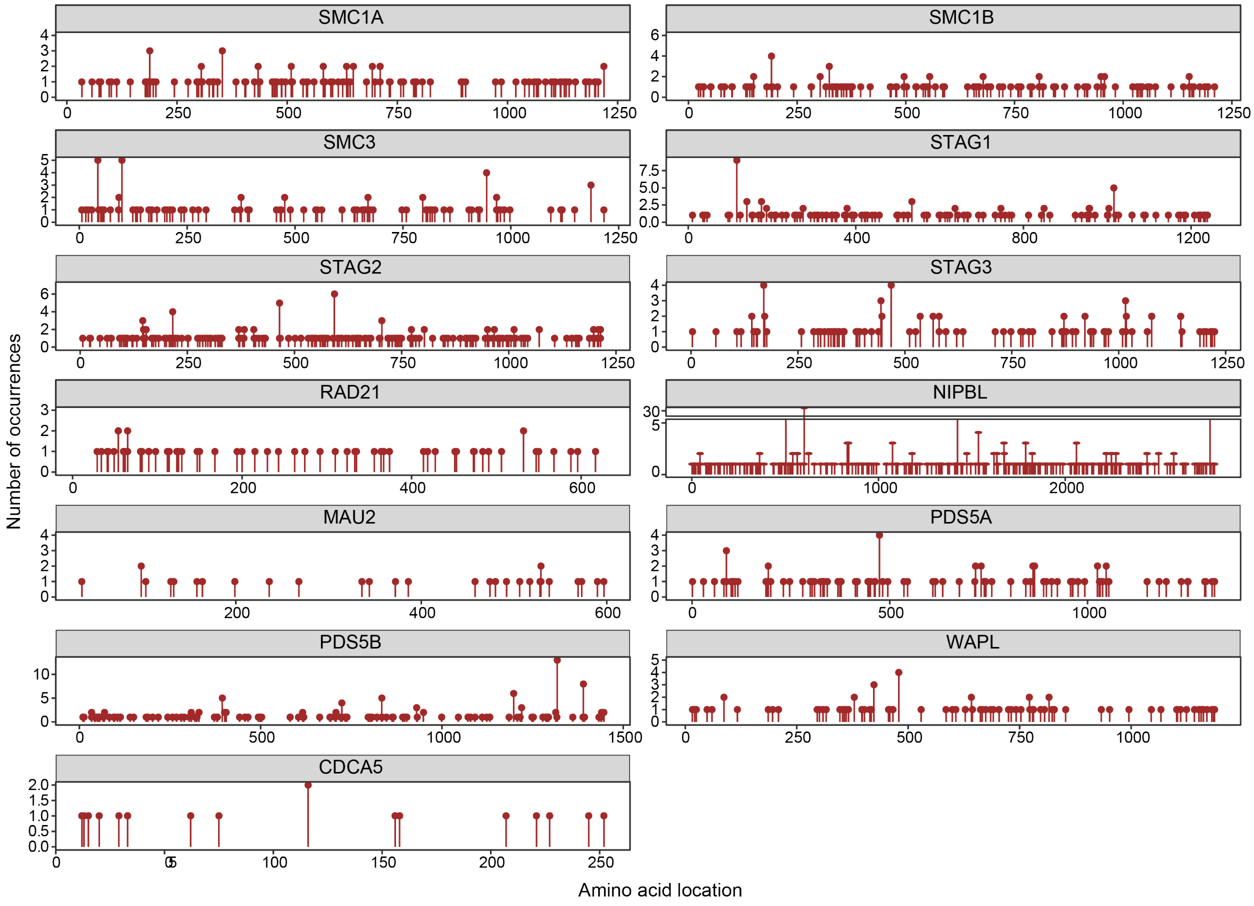

Supplemental Figure 3. Spectra of cohesin SmV in cancer. Lollipop charts depicting frequency of deleterious SmV found in cohesin complex genes in TCGA exomes.

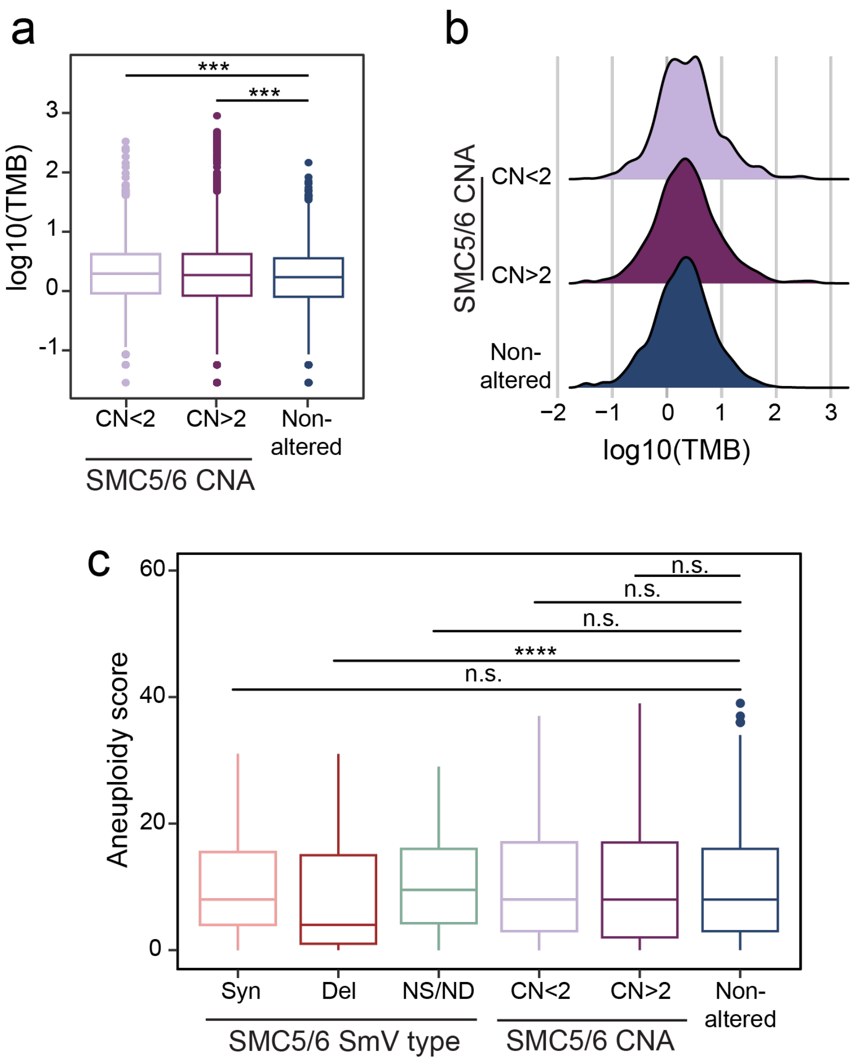

Supplemental Figure 4. SMC5/6 SmV do not impact tumor aneuploidy. TMB in cohorts of TCGA tumors with copy number alterations in SMC5/6 genes shown as (**a**) box and whisker plot, and (**b**) ridge line plot demonstrating similar TMB (median range 2-2.3 mutations per Mb) between the compared groups. (**c**) Aneuploidy score of TCGA tumor cohorts categorized by type of SmV or copy number alteration in SMC5/6 genes. Significance determined by Wilcoxon rank-sum test; **** $p<0.0001$.

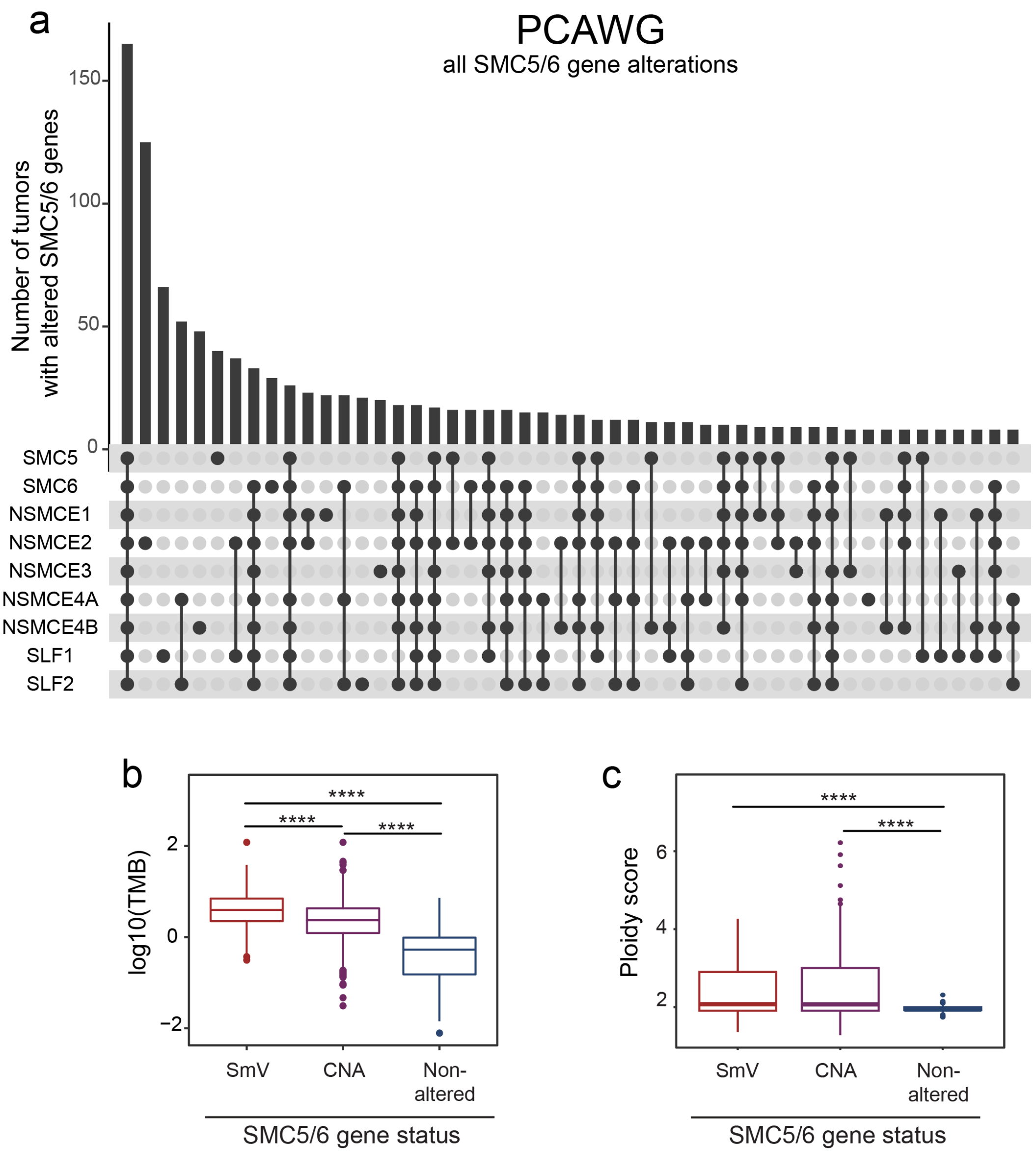

Supplemental Figure 5. Elevated TMB associated with deleterious SmV in SMC5/6 genes in PCAWG genomes. (**a**) UpSet plot depicting frequency of SMC5/6 alterations (exonic SmV, low, and high CNV) in tumors from PCAWG. (**b**) TMB in PCAWG cohorts of genomes with SmV, CNA, or non-altered SMC5/6 genes, and (**c**) ploidy score indicating degree of aneuploidy (median values range 1.94-2.08 chromosome sets per cell). Significance determined by Wilcoxon rank-sum test; **** p<0.0001

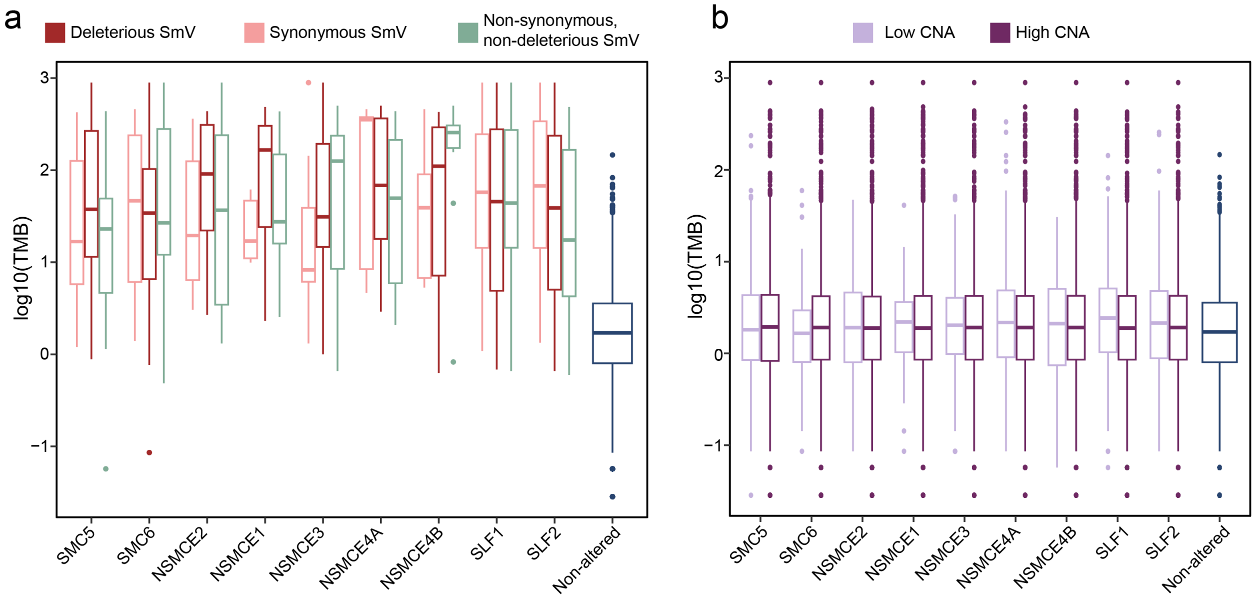

Supplemental Figure 6. Variants in all SMC5/6 genes are associated with increased TMB. TMB in cohorts of TCGA tumors separated by type of SmV (**a**) or degree of copy number alteration (**b**) in each SMC5/6 gene.

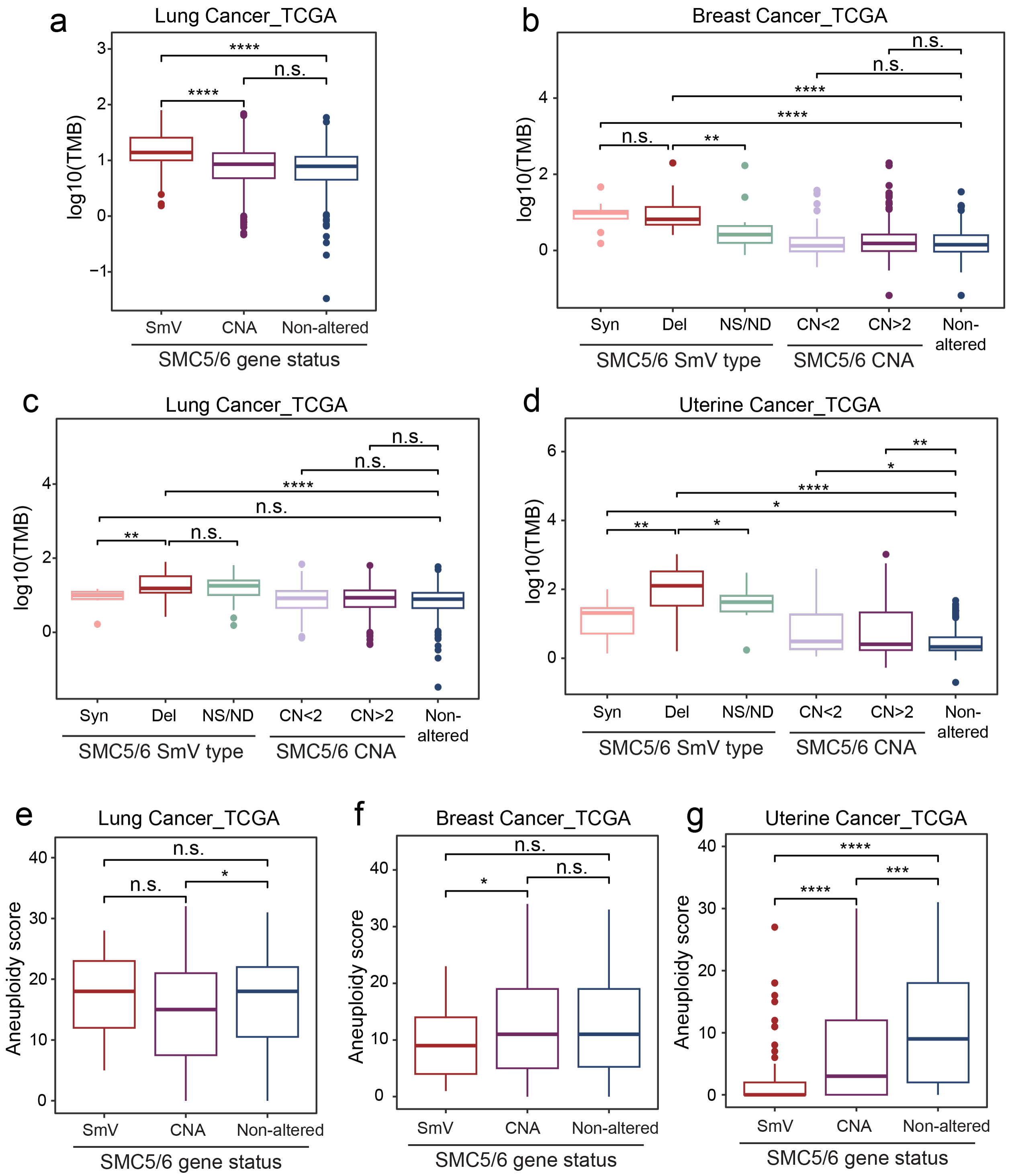

Supplemental Figure 7. SMC5/6 variants correlate with elevated TMB in disease-specific cohorts. (**a**) TMB in the TCGA lung cancer cohort of genomes with SmV, CNA, or non-altered SMC5/6 genes, (**b-d**) TMB in disease-specific cohorts of genomes categorized by type of SmV or copy number alteration in SMC5/6 genes. (**e-g**) Aneuploidy score indicating degree of aneuploidy in tumor genomes categorized by SmV, CAN, or non-altered SMC5/6 genes from lung (e), breast (f), and uterine (g) cohorts. Significance determined by Wilcoxon rank-sum test; *p<0.05, **p<0.01, **** p<0.0001.

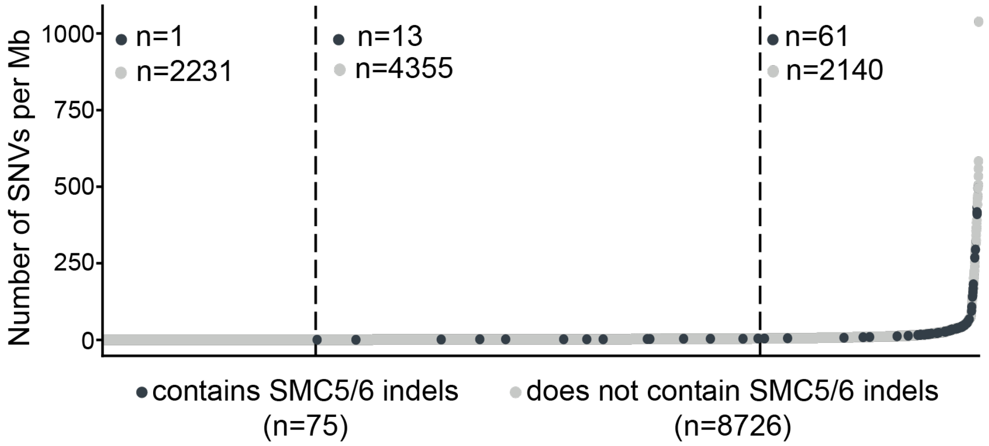

Supplemental Figure 8. SMC5/6 gene disruption by indel is associated with elevated burden of single nucleotide variants (SNV). SNV burden (excluding indels) of each tumor exome in TCGA is plotted from lowest to highest. Dark gray dots designate tumors with SMC5/6 gene indels and gray dots are all other tumors. Dotted lines denote lower and upper quartiles. Number of each type of tumor per section is shown. X^2 test for independence: 129.08, df=2, p<2.2e-16.

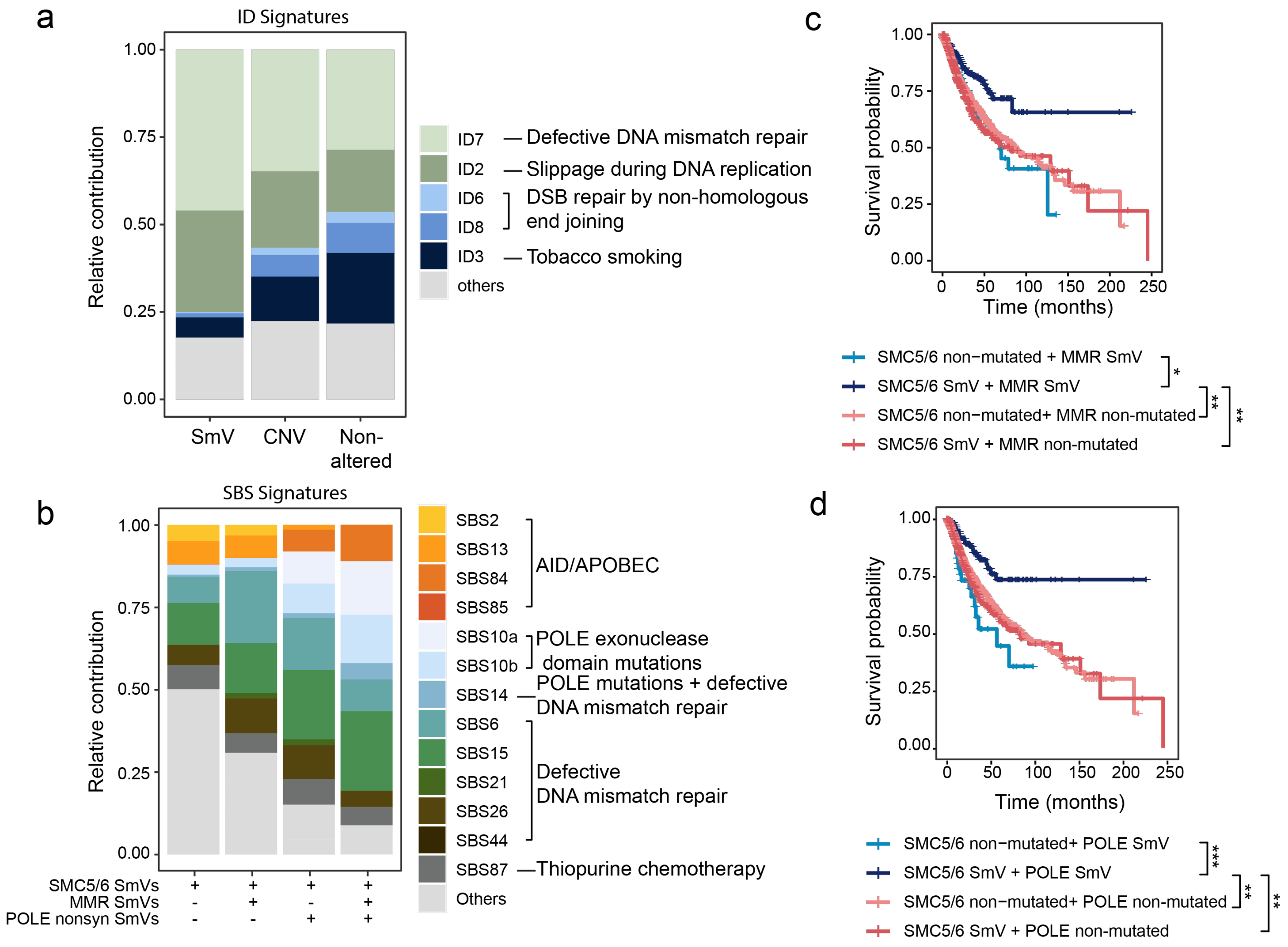

Supplemental Figure 9. SMC5/6 SmV in combination with POLE or MMR SmV augment survival outcomes. (**a**) Relative contribution of indel (ID) signatures from TCGA exomes categorized by presence of SMC5/6 gene alteration relative to those with non-altered SMC5/6 genes. Etiology of ID signatures is shown in legend. ID signatures that contribute <5% of total in analyzed exomes are designated "others". (**b**) Relative contribution of single base substitution (SBS) signatures from TCGA exomes categorized by nonsynonymous SmV in SMC5/6 complex genes and MMR genes and/or POLE. Etiology of SBS signatures is shown in legend. Colored signatures are designated with etiology in legend; these signatures comprised >5% relative contribution of TMB in SMC5/6 deleterious SmV cohort. Signatures in gray are designated "others" and comprised <5% relative contribution of TMB in deleterious SmV cohort (**c-d**) Kaplan-Meier curve of TCGA exomes with non-synonymous SmV in SMC5/6 genes, MMR genes, and/or POLE. p-value determined by log-rank test.
